# Characterizing genetic variation on the Z chromosome in *Schistosoma japonicum* reveals host-parasite co-evolution

**DOI:** 10.1186/s13071-024-06250-4

**Published:** 2024-05-08

**Authors:** An Zhou, Wei Zhang, Xueling Ge, Qi Liu, Fang Luo, Shuhua Xu, Wei Hu, Yan Lu

**Affiliations:** 1 State Key Laboratory of Genetic Engineering, Center for Evolutionary Biology, School of Life Sciences, Fudan University, Shanghai, 200438 China; 2 Ministry of Education Key Laboratory of Contemporary Anthropology, Collaborative Innovation Center for Genetics and Development, Fudan University, Shanghai, China; 3 Key Laboratory of Computational Biology, Shanghai Institute of Nutrition and Health, University of Chinese Academy of Sciences, Chinese Academy of Sciences, Shanghai, 200031 China; 4 Human Phenome Institute, Zhangjiang Fudan International Innovation Center, and Ministry of Education Key Laboratory of Contemporary Anthropology, Fudan University, Shanghai, 201203 China; 5 School of Life Science and Technology, Shanghai Tech University, Shanghai, 201210 China; 6 College of Life Sciences, Inner Mongolia University, Hohhot, 010070 China

**Keywords:** *Schistosoma japonicum*, Z chromosome, Genetic diversity, Adaptive evolution

## Abstract

**Background:**

Schistosomiasis is a neglected tropical disease that afflicts millions of people worldwide; it is caused by Schistosoma, the only dioecious flukes with ZW systems. *Schistosoma japonicum* is endemic to Asia; the Z chromosome of *S. japonicum* comprises one-quarter of the entire genome. Detection of positive selection using resequencing data to understand adaptive evolution has been applied to a variety of pathogens, including *S. japonicum*. However, the contribution of the Z chromosome to evolution and adaptation is often neglected.

**Methods:**

We obtained 1,077,526 high-quality SNPs on the Z chromosome in 72 *S. japonicum* using re-sequencing data publicly. To examine the faster Z effect, we compared the sequence divergence of *S. japonicum* with two closely related species, *Schistosoma haematobium* and *S. mansoni*. Genetic diversity was compared between the Z chromosome and autosomes in *S. japonicum* by calculating the nucleotide diversity (π) and Dxy values. Population structure was also assessed based on PCA and structure analysis. Besides, we employed multiple methods including Tajima’s *D*, F_ST_, iHS, XP-EHH, and CMS to detect positive selection signals on the Z chromosome. Further RNAi knockdown experiments were performed to investigate the potential biological functions of the candidate genes.

**Results:**

Our study found that the Z chromosome of *S. japonicum* showed faster evolution and more pronounced genetic divergence than autosomes, although the effect may be smaller than the variation among genes. Compared with autosomes, the Z chromosome in *S. japonicum* had a more pronounced genetic divergence of sub-populations. Notably, we identified a set of candidate genes associated with host-parasite co-evolution. In particular, *LCAT* exhibited significant selection signals within the Taiwan population. Further RNA interference experiments suggested that *LCAT* is necessary for *S. japonicum* survival and propagation in the definitive host. In addition, we identified several genes related to the specificity of the intermediate host in the C-M population, including *Rab6* and *VCP*, which are involved in adaptive immune evasion to the host.

**Conclusions:**

Our study provides valuable insights into the adaptive evolution of the Z chromosome in *S. japonicum* and further advances our understanding of the co-evolution of this medically important parasite and its hosts.

**Graphical Abstract:**

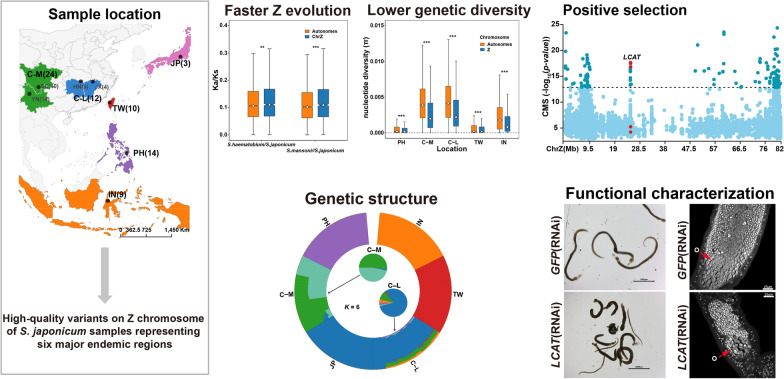

**Supplementary Information:**

The online version contains supplementary material available at 10.1186/s13071-024-06250-4.

## Background

Schistosomiasis is a devastating parasitic disease, second in importance only to malaria. It is caused by *Schistosoma* and affects at least 251.4 million people in 78 countries [1, 2]. *Schistosoma japonicum* is a dioecious trematode (fluke) mainly distributed in Asian countries, including China, the Philippines, Japan, and Indonesia [3, 4]. *Schistosoma japonicum* has a certain degree of local adaptability because it needs to complete its parasitic life cycle in specific environments within the host’s body. The life cycle of *S. japonicum* is complex. *Schistosoma japonicum* infects *Oncomelania hupensis*, a freshwater snail, as the sole intermediate host and exhibits a broad definitive host range, infecting more than 46 mammals, including humans [5, 6]. There are different reproduction modes within different host stages: asexual reproduction in freshwater snails and sexual reproduction in mammalian definitive hosts. Eggs produced by worm pairs in the definitive host are the primary pathogenic factor, which can cause mechanical damage and complex immunopathological responses in the host. Currently, only one drug is available (praziquantel) for treatment.

Studies on morphology, microsatellite markers, mitochondrial DNA, and autosomes have unveiled distinct subpopulations within *S. japonicum* [7–11]. The parasites from TW (Taiwan Province, China), PH (the Philippines), and IN (Indonesia) have significant genetic differentiation due to geographical isolation. Populations of *S. japonicum* show many pronounced phenotypic differences. For example, the Taiwan strain cannot parasitically reproduce in humans; it exhibits zoophilia and has weaker pathogenicity [5]. Regarding the intermediate hosts, it is difficult for the Chinese lake strain to parasitize snails in the mountainous areas of China [12, 13]. Local adaptation of schistosomes has always been a focal area of parasitic research, drawing persistent attention. For instance, *S. mansoni* may develop resistance to praziquantel because of prolonged exposure to mass drugs [14, 15]. Genome-wide variations were also used to detect local adaptation evidence in *S. japonicum*, which may be involved in host-switching [16]. However, the sex chromosomes were disregarded. Compared with autosomes, sex chromosomes have smaller population size (*Ne*). The degeneration of the Y or W chromosome is thought to be mainly attributed to the lack of recombination, leading to the loss of ancestral genes [17, 18]. Due to hemizygosity in one sex gender, new mutations on the X and Z chromosomes may be more exposed to selection than those on the autosomes [19, 20]. This is one of the reasons leading to a faster molecular evolution rate on the X/Z chromosomes (known as faster-Z or faster-X effect) compared to autosomes, while another reason is increased genetic drift [20, 21]. The Z chromosome of *Schistosoma* contributes approximately 20% of the total genome size. According to the latest genome assembly (SM_V10) of *S. mansoni* [22], the Z chromosome contributes ~ 22.1%. For *S. japonicum* [23], this contribution is estimated at 21.4%, while for *Schistosoma haematobium* [24], it is about 22.3% (with a mixed ZW assembly). *Schistosoma bovis* and *S. mekongi* exhibit contributions of 21.6% and 22.8%, respectively [25, 26]. Genes located on the Z chromosome may be involved in signal transduction associated with environmental information, progesterone-mediated oocyte maturation, cell cycle, and other important processes [24, 26]. Currently, there is a lack of empirical evidence indicating a faster evolutionary rate on the Z chromosome in schistosomes, and the potential contribution of Z chromosomes to local adaptation remains unclear.

For the within-species Z chromosome variation dataset of *S. japonicum*, we obtained 1,077,526 high-quality single-nucleotide polymorphisms (SNPs) on the Z chromosome from 72 wild male individuals, as reported by Luo et al. [16]. We performed the selection scans based on these SNPs to detect signals of positive selection. By comparing sequence divergence between *S. japonicum* and both *S. mansoni* and *S. haematobium*, we found a faster evolution rate of the Z chromosome compared to the autosomes. Furthermore, we identified some local adaptation evidence that may be associated with host fitness.

## Methods

### Data collection and alignment

The re-sequenced data of 72 male *S. japonicum* samples were downloaded from the Genome Sequence Archive database in NCBI under accession number PRJNA789681 [16], including 11 from the Philippines (PH), 9 from Indonesia (ID), 3 from Japan (JP), 10 from Taiwan Province, China (TW), 14 from the China low-lying lakes (C-L), and 24 from the China mountainous regions (C-M). Five *S. mansoni* individuals’ resequencing data were downloaded from European Nucleotide Archive (ENA) (SRA accession numbers: SRR13624153, SRR13624155, SRR13624156, SRR13624157, and SRR13624158) [27]. The genomic data sets of *S. haematobium* and *S. mansoni* were download from WormBase ParaSite (https://parasite.wormbase.org/index.html) with accession numbers PRJNA78265 [28] and PRJEA36577 [22], respectively. The genomic data of *S. japonicum* was download from NCBI with accession number PRJNA739049 [16].

### Variant calling and filtering

Raw reads in fastq format were quality trimmed and filtered using fastp (v0.23.1) [29]. The cleaned reads were aligned to the *S. japonicum* chromosome-level reference genome (SjaV3) [16] using BWA mem (Version: 0.7.17) [30] with the parameter “-M”. Samtools (version 1.14) [31] was used to separate and sort the mapped reads and finally produce a final binary alignment map (bam) for each individual. Picard was used to mark and remove duplicates based on the resulting BAM file. Subsequent data processing was almost completely implemented with GATK [31].

Due to the lack of known whole-genome variable site databases, we used the method recommended on the GATK website for non-human data and proceeded as follows: An initial round of SNP and InDel calling was performed by GATK HaplotypeCaller for each sample; we first obtained the genomic variant call format (GVCF), then used GATK CombineGVCFs to combine the GVCF files, and subsequently performed joint-call cohort genotyping using GATK GenotypeGVCFs. We separated single-nucleotide polymorphisms (SNPs) and IndDels with GATK SelectVariants and subsequently performed hard filtering using GATK VariantFiltration with default criteria (for SNPs: QD < 2.0, FS > 60.0, MQ < 40.0, MQRankSum <  − 12.5, ReadPosRankSum <  − 8.0; for short InDels: QD < 2.0, FS > 200.0, SOR > 10.0, MQRankSum <  − 12.5, ReadPosRankSum <  − 20.0). The remaining SNPs and InDels after the above steps were utilized as the true-positive variant dataset for Base Quality Score Recalibration (BQSR). SNP and InDel calling, joint-call cohort genotyping, and hard filtering were repeated based on the recalibrated BAM files generated by GATK. To further improve the quality of the SNP dataset, we masked variants located in repeat regions using the third quartile of SNP density in non-transposable element (TE) regions as a threshold [16]. In this study, we focused on the variation on the Z chromosome. To minimize false positives for SNPs, we filtered out the SNP sites with a missing rate (–max-missing) > 10% and minor allele frequency (MAF) < 5% through Vcftools (version 0.1.13) [32]. Additionally, only biallelic sites were retained. Functional annotation of SNPs was performed according to the reference genome (SjaV3) [16] using ANNOVAR (version 2015-12-14) [33]. A total of 1,077,526 SNPs on the Z chromosome remained. We phased these variants using Beagle (version 5.2) [34]. We used KING [35] to judge the kinship among all individuals based on the autosomal SNPs.

We performed CDS sequence alignments by diamond (v2.1.8.162) in a pairwise manner between *S. japonicum* and *S. haematobium* and between *S. japonicum* and *S. mansoni*; then, nonsynonymous (Ka) and synonymous (Ks) substitution rates (Ka/Ks) were calculated by KaKs_Calculator v3.0 [36]. We calculated adaptive substitution rate (α) by Z chromosome by McDonald-Kreitman (MK) test using PopGenome (R package) [37, 38]. The synonymous fixed divergence (dS) and nonsynonymous fixed divergence (dN) were calculated between *S. japonicum* and *S. mansoni*; the numbers of synonymous polymorphisms (*P*_s_) and non-synonymous polymorphisms (*P*_n_) were calculated within each *S. japonicum* population. We tested for differences between Z and autosomes using the Mann-Whitney-Wilcoxon test (the pairwise equivalent of the Kruskal-Wallis test) with R.

### Population structure analysis

SNPs for population structure analysis were analyzed using PLINK (version 1.90b6.24) [39] to remove linkage disequilibrium (LD) with the parameters “-indep-pairphase 100 10 0.2.” We obtained 44,829 variants on the Z chromosome that were not in linkage disequilibrium. We performed principal component analysis (PCA) and neighbor-joining (NJ) phylogenetic analysis by plink [39] with the parameters “–pca” and “–distance 1-ibs,” respectively. The results of the principal component analysis (PCA) were visualized via R (version 3.6.0), and we then imported the resulting identity-by-state distance matrix into MEGA (https://www.megasoftware.net/) to produce a neighbor-joining phylogenetic tree without an outgroup. Proportions of individual ancestry were inferred using ADMIXTURE (Version 1.3.0) [40] with the parameters “-cv -j4”, with *K* values (number of hypothetical ancestral populations) ranging from 1 to 10 and repeated 10 times per K value with different random seeds. The cross-validation error (CV) value was calculated for each *K* to determine the most supported value. The lowest cross-validation error (CV) value was found for *K* = 6. The run results were imported into CLUMPAK [41] for integrated clustering and visualization. AncestryPainter2 [42] was used to visualize the results for the optimal *K* value (*K* = 6).

### Genetic diversity analysis

Genetic diversity analysis was performed with 5,547,761 high-quality SNPs of autosomes and 1,077,526 high-quality SNPs of the Z chromosome. Nucleotide diversity (π) was calculated for both autosomes and the Z chromosome by VCFtools (Version 0.1.13) [32] in 10-kb sliding and 5-kb overlapping step windows. We retained the sliding windows that contained two or more SNPs. The results were visualized using R (version 4.2.0). Absolute divergence (Dxy) was calculated by pixy (version 1.2.7.beta1) based on a window of 10 kb [43].

### Detection of selection signals by scanning the Z chromosome

To evaluate the evolutionary response to selection, we employed multiple methods to detect selection signals based on 1,077,526 phased bi-allelic SNPs of the Z chromosome. Tajima’s *D* and integrated haplotype scores (iHS) were used to detect selected signals of SNPs within the TW and C-M populations. The fixation index (F_ST_) and cross-population extended haplotype homozygosity (XP-EHH) were used to detect the positive selection genomic signatures between lake strains and others (TW and C-M). We calculated Tajima’s *D* by a publicly available Python script [44] (https://github.com/Shuhua-Group/Theta_D_H.Est) in 10-kb sliding windows using a 5-kb step size. IHS and XP-EHH were calculated for each SNP with the rehh [45]. F_ST_ was calculated by VCFtools (v0.1.13) [32] in the same sliding windows mentioned above. Negative F_ST_ values were corrected to 0, and then the values were standardized and transformed into z-scores. For different tests, we used different strategies to identify empirical thresholds and to calculate scores for each window. For F_ST_ and Tajima’s *D*, we chose the top 1% of the distributions as empirical thresholds. For iHS and XP-EHH, we chose the top 0.1% and top 0.5% of the distributions, respectively, as empirical thresholds. SNPs with values above the empirical thresholds were considered significant SNPs in each test. For defining candidate windows and calculating the scores of each window (window score), F_ST_ and Tajima’s *D* were already window-based calculations. For XP-EHH and iHS, we first divided the Z chromosome into 10-kb windows with 5-kb overlap. Windows containing two or more significant SNPs were identified as selected genomic regions, and the mean significance value within this window was calculated as the window score [46]. A composite of multiple signals (CMS) was used to generate a composite score for the TW population based on the results of the four independent methods mentioned above. Before calculating CMS, we processed the results obtained by the above four methods as follows. We calculated F_ST_ and Tajima’s *D* for each SNP site on the Z chromosome by Vcftools (version 0.1.13) [32]. Negative F_ST_ values were corrected to 0. We removed values < 0 from the XP-EHH results and obtained the absolute values of Tajima’s *D* and iHS. We finally conducted a CMS analysis using estimates of the above four statistics as inputs using a method described in a previous study [47, 48]. SNPs with the highest 1% CMS scores were identified as selection signals. The method used to identify candidate windows for CMS was the same as for the XP-EHH and iHS tests. PBScan (version 2020.03.16) was used to calculate the population branching statistic (PBS) [49].

Finally, all candidate selection regions selected by the above methods were annotated by BEDOPS (version 2.4.40) [50]. We then performed gene ontology analysis with the clusterProfiler [51] for selected genes.

### Differential gene expression analysis

Female and male RNA-seq data for 14–28 dpi were downloaded from the NCBI database (project number PRJNA343582) [52], and the RNA-seq data of the four developmental stages (egg, miracidium, sporocyst, and cercaria) of *S. japonicum* larvae were obtained from NCBI (project ID: PRJNA719283) [53]. We used fastp (version 0.23.1) [29] to perform quality control on the raw data. The clean reads were mapped to the chromosome-level reference genome of *S. japonicum* (SjaV3) by HISAT2 (version 2.2.1) [54] and then used HTSeq [55] to convert the comparison results into gene-level counting matrices. Finally, edgeR (version 3.38.2) [56] was used for data normalization and expression estimation, and normalization was performed by the transcript per million (TPM) method [57].

### Functional experiments for the target genes

To explore the functions of *LCAT, Rab6*, and *VCP*, we used double-stranded RNA (dsRNA) to interfere with 30-day-old paired adult worms and observed them in vitro. Two primers for the dsRNA template were generated by PCR, each approximately 500 base pairs in length and containing the T7 promoter sequence. The dsRNA was prepared and purified using a MEGAscript T7 transcription kit (Ambion; Foster City, CA, USA) according to the manufacturer’s instructions. Non-specific green fluorescent protein (*GFP*) dsRNA was prepared as a negative control. For RNAi in vitro, mice were infected with 200 cercariae by the abdominal patch method, and on the 30th day after infection, the mouse hepatic portal vein was perfused with sterile saline containing sodium heparin to obtain mature *S. japonicum*. We then selected pairs of worms and placed them in 12-well culture plates for in vitro culture, six pairs of worms per well, and added 3.5 ml DMEM medium containing 10% FBS, 200 μM ascorbic acid, and 0.2% V/V mouse red blood cells. We added 10 μg/ml dsRNA to the medium on the 1st, 3rd, 5th, and 7th days and then added dsRNA every 4 days. After 14 days of dsRNA treatment, gene knockdown was monitored by qRT-PCR. The statistical analysis of knockdown levels of gene transcription using Welch’s *t*-test was performed in the GraphPad Prism program (GraphPad Software). The remaining worms were immediately fixed in alcohol-formalin-acetic acid (AFA) and stained with Mayer’s carmalum as described in a previous study [58]. We used confocal laser scanning microscopy to study the morphology of the reproductive organs of the parasites.

## Results

### Lower genetic diversity and faster evolution of the Z chromosome in *S. japonicum* compared with autosomes

**Fig. 1:**
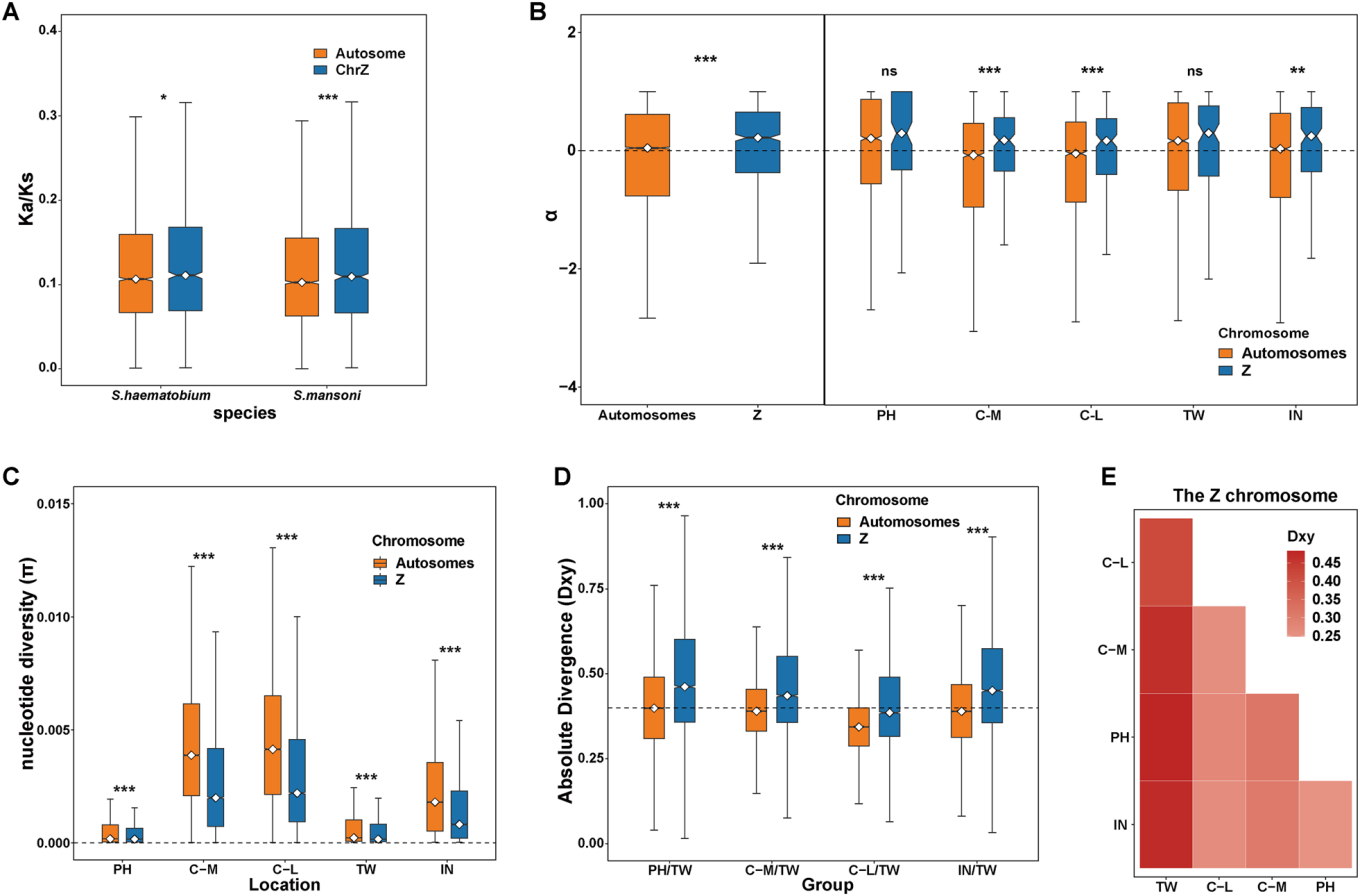
Population genetic parameters across the genomes of *Schistosoma japonicum* to detect the supporting evidence for faster and more adaptive Z chromosome evolution. Mann-Whitney test was used for significant differences between the Z and autosomes denoted by *P < 0.05, **P < 0.01, ***P < 0.001. **A** Sequence divergence was compared in a pairwise manner between *S evidence japonicum* and *S. haematobium* and between *S. japonicum* and *S. mansoni*. **B** Adaptive substitutions rate (α) across the genomes of *S. japonicum*. Compare the adaptive substitution rate of Z chromosome and autosomes using all *S. japonicum* individuals (left of dash). Compare the adaptive substitution rate of the Z chromosome and autosomes for each population of *S. japonicum* (right of dash). **C** Nucleotide diversity (π) in five *S. japonicum* sub-populations calculated based on variation of the Z chromosome and autosomes. **D** Boxplot of absolute divergence (Dxy) between TW and other subpopulations. The dotted line represents a Dxy value of 0.4. **E** Heatmap of absolute divergence (Dxy) between each pair of subgroups for the sex chromosome (Z chromosome). The darker the red color, the higher the value

Based on 6717 *S. japonicum*/*S.haematobium* alignments and 7225 *S. japonicum*/*S. mansoni*, we calculated the ratio of nonsynonymous to synonymous substitution rates (Ka/Ks or dN/dS) (see Methods). The median Ka/Ks of the Z chromosome is significantly higher than that of the autosomes (*S. japonicum* versus *S. haematobium*, W = 3,876,423, *P* = 0.02006; *S. japonicum* versus *S. mansoni*, W = 4,384,558, *P* = 3.34 × 10^–4^), indicating a faster evolution rate on Z chromosome (Fig. 1A; Additional file 2: Table S1). When considering the variation among genes, the effect of genetic divergence may not be substantial. To examine rates of adaptation, we calculated the proportion of adaptive substitutions (α) with the McDonald-Kreitman (MK) test [37, 59] with *S. mansoni* as outgroup. We first examined α for both the Z chromosome and autosomes using all *S. japonicum* samples and then further split samples by populations. With all samples, the Z chromosome showed significantly higher rates of adaptive evolution (α) than the autosomes (*W* = 6,978,402, *P* < 0.0001) (Fig. 1B; Additional file 2: Table S2). Partitioning samples by populations, consistent results were observed across each population subgroup. Elevated α values were consistently observed on the Z chromosome in all subpopulations; even the differences in the TW and PH populations were not statistically significant (Fig. 1B; Additional file 2: Table S2). The nucleotide diversity (π) was also significantly lower (*P* < 0.0001) on the Z chromosome than on the autosomes (Fig. 1C; Additional file 2: Table S3). These results support faster and more adaptive evolution on the Z chromosome than autosomes in *S. japonicum* [60, 61]. Among the diverse subpopulations of *S. japonicum*, TW population showed significantly lower π values than other populations on both Z chromosome and autosomes (Fig. 1C; Additional file 1: Fig. S1A; Additional file 2: Table S3). Furthermore, we calculated the absolute divergence (Dxy) between each pair of subpopulations and found that the TW population exhibited a greater degree of genetic differentiation than other subgroups, followed by the C-M population (Fig. 1E; Additional file 1: Fig. S1B). Notably, a higher Dxy value for the Z chromosome compared to the autosomes (Fig. 1D; Additional file 1: Fig. S1B) suggests significant variation in genetic differentiation between autosomes and sex chromosomes in *S. japonicum* [62]. Overall, the largest absolute divergence (Dxy) and the lowest nucleotide diversity (π) in the Z chromosome of the TW population indicated potential local adaptation.

### Population structure revealed by the Z chromosome of *S. japonicum*

**Fig. 2:**
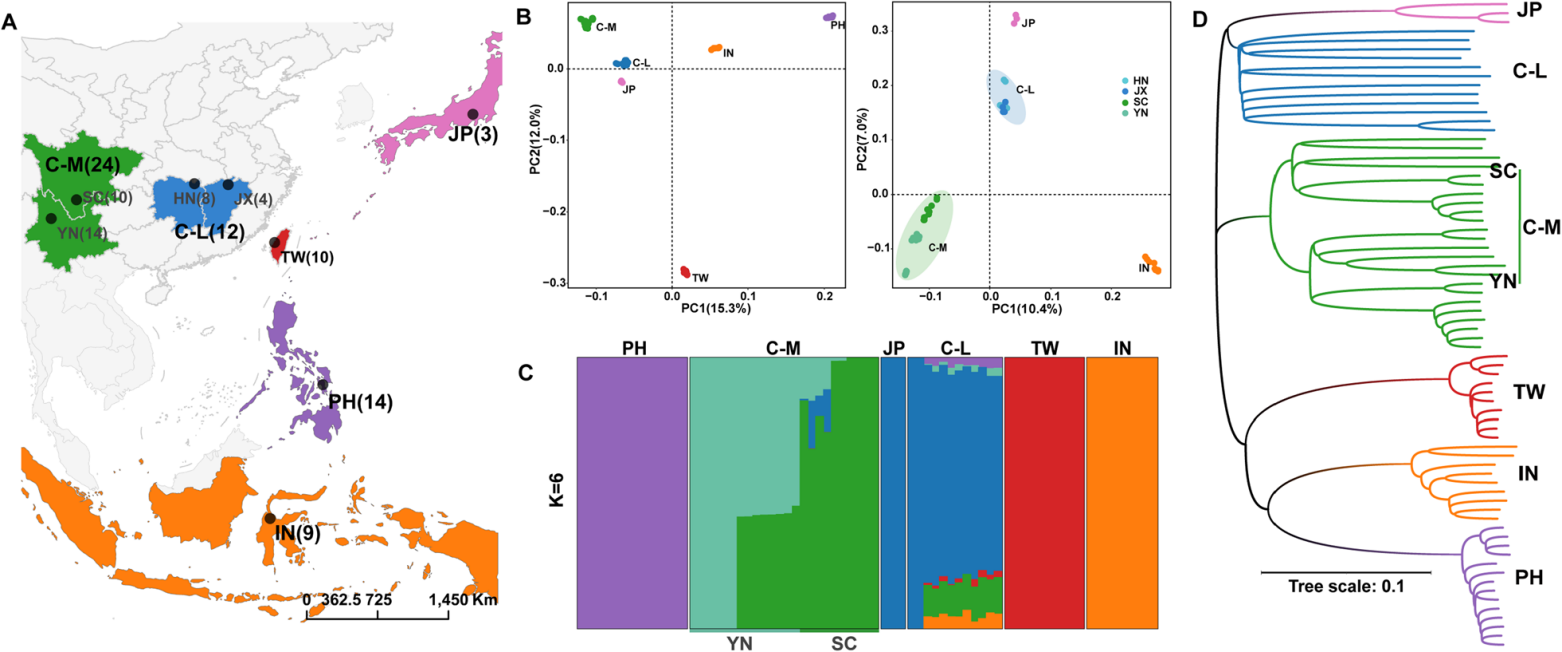
Genetic diversity and population structure of sampled *Schistosoma japonicum*. **A** Map of sample collection for *S. japonicum*. **B** Principal component analysis (PCA) of *S. japonicum* male adults. The graph on the left is based on the genetic variation from all individuals’ Z chromosomes; the graph on the right is based on other individuals except TW (10) and PH (14). **C** Population structure of 72 *S. japonicum* individuals based on their Z chromosome variants. **D** Phylogenetic tree constructed based on the Z chromosome variations of 72 *S. japonicum* samples

Resequencing data of Z chromosomes from 72 male *S. japonicum* individuals across their main distribution areas were selected for further analysis (Fig. 2A). We identified 1,077,526 high-quality SNPs on the Z chromosome, with an average 15 × depth based on a mapping to a chromosome-level *S. japonicum* reference genome. Principal component analysis (PCA) revealed that *S. japonicum* individuals were divided into six distinct subclades, indicating a regional distribution pattern (Fig. 2B). Substructure of the Z chromosome was also observed in mountain populations (Fig. 2C). A neighbor-joining (NJ) phylogenetic tree including six distinctly independent clades produced the same findings as the PCA (Fig. 2D). Assuming *K* = 6, we got the lowest cross-validation error (Additional file 1: Fig. S2B) and discovered that three island regions including PH (the Philippines), TW (Taiwan province, China), and IN (Indonesia) shared no genetic components, like in preview studies [10, 16], possibly as a result of geographic isolation (Fig. 2C). Notably, based on genetic variation on the Z chromosome, Chinese mainland populations (mountain and lake) showed different genetic components from a previous study based on autosomes [16]; samples from Sichuan and Yunnan in the C-M region (China mountainous regions) also possessed different genetic components, which supports further substructure within the C-M population (Fig. 2C). Our results were consistent with previous studies using mitochondrial DNA [10] and autosome [16] data demonstrating that *S. japonicum* from Taiwan, the Philippines, Indonesia, Japan, and the Chinese lake and mountain populations exhibited evident geographical distribution patterns. There has been almost no gene exchange between the island locations due to long-term geographical isolation. The TW population had the highest degree of genetic differentiation, whereas the lake population had more complex ancestral components. Interestingly, the population substructure in the mountain population as revealed by the *S. japonicum* Z chromosome was more pronounced (Fig. 2C).

### Genomic signatures of natural selection in the Z chromosomes of the Taiwan (TW) population

**Fig. 3:**
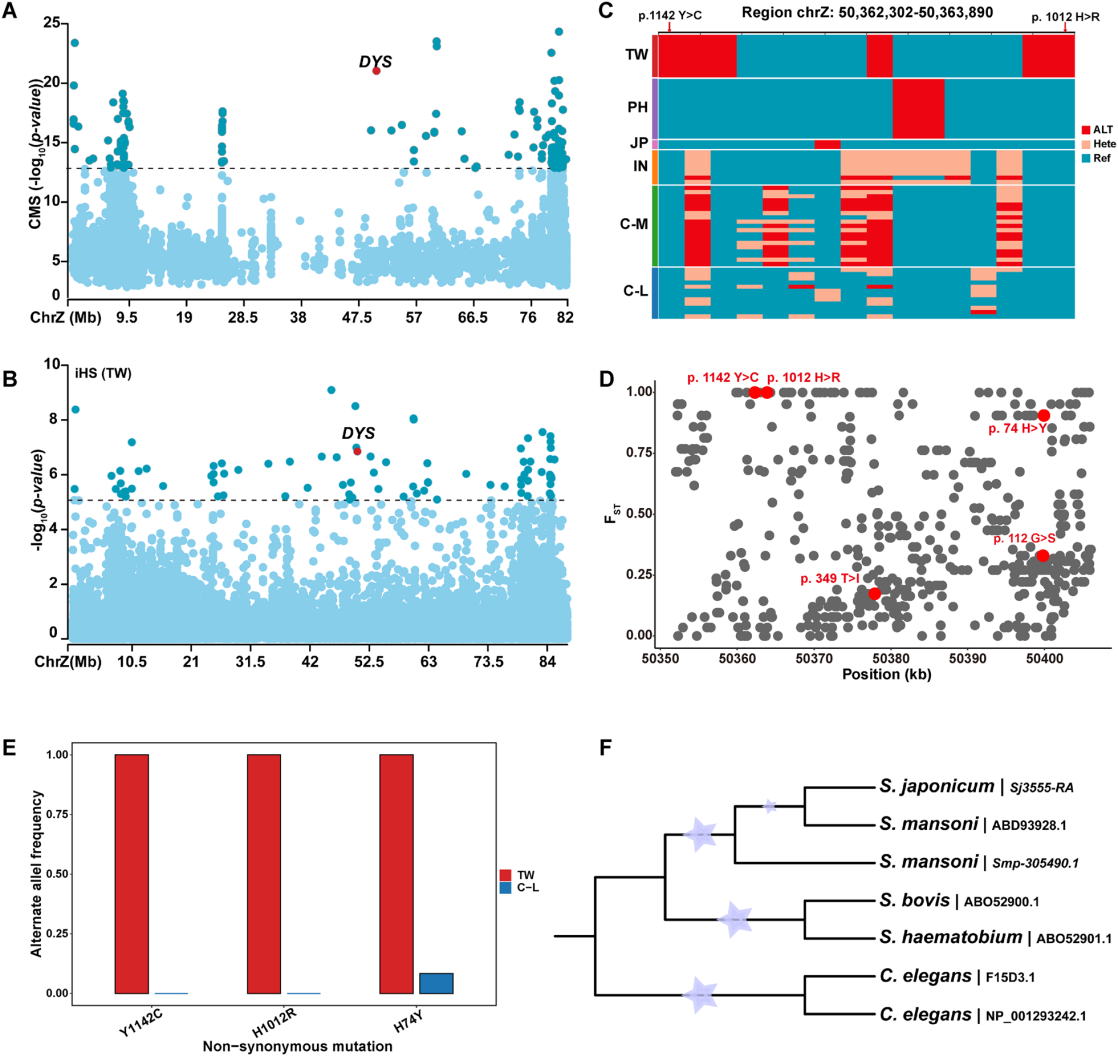
Positively selected gene *DYS* in TW population identified by CMS and iHS. **A** and **B** Positively selected signatures of *DYS* (*Sj3555*) from the CMS and iHS analysis. The dashed lines represent the empirical thresholds for the selected region (top 0.1% empirical distribution of *P*-value of iHS test = 5.0169; top 1% empirical distribution of CMS score = 12.831). The genome region harboring *DYS* (dystrophin) is marked in the figure. **C** Haplotype heatmap for SNP variants within the *DYS* gene region. Two nonsynonymous variants are marked with arrows. **D** F_ST_ distribution in the *DYS* gene region (ChrZ: 50351736–50406101). Five nonsynonymous variants within the TW version *DYS* are highlighted in red. **E** Alternative allele frequency for three non-synonymous mutations in the TW and C-L populations. **F** NJ phylogenetic tree based on the dystrophin full-length or C-terminal protein sequence. *Caenorhabditis elegans* was used as the outgroup

The *S. japonicum* Z chromosome contains a total of 2116 genes involved in a variety of important biological processes, including DNA damage repair, sensory system development, and fatty acid derivative metabolic processes (Additional file 1: Fig. S3, Additional file 2: Table S4). To better understand the genomic features of the Z chromosomes of the TW population, we performed selective sweep analyses (fixation index: F_ST_, integrated haplotype score: iHS, cross-population extended haplotype homozygosity: XP-EHH, and Tajima’s *D*; see Methods) to identify candidate genes involved in different host environments. In addition, we used the Composite of Multiple Signals (CMS) statistics to combine the signals detected in the four approaches mentioned above. According to the CMS score, we identified 36 candidate regions encompassing 34 candidate genes that exhibited significant signals (Fig. 3A and Additional file 2: Table S5). Functional analysis showed that these genes were significantly enriched for GO terms related to the regulation of the cell proliferation process (GO:0010972, *P* = 4.26 × 10^–3^; GO:1,902,750, *P* = 4.99 × 10^–3^), lipoprotein metabolic process (GO:0042157,* P* = 1.55 × 10^–2^), and sensory perception (GO:0007605, *P* = 1.15 × 10^–2^), all of which are important biological processes (Additional file 2: Table S6).

Among these 34 candidate genes, we found that the *DYS* gene (*Sj3555*) had a pronounced signature of positive selection (Fig. 3A and B). The region ChrZ: 50,395,000–50,410,000 covering the *DYS* (*Sj3555*) gene showed the highest window score (CMS value = 21.040, Additional file 2: Table S5; see Methods) as well as the largest iHS (maximum |iHS| value = 5.265). The *DYS* gene encodes the dystrophin protein, which is recognized as the founding member of a protein superfamily with representatives throughout the animal kingdom [63]. Mutations in the *Caenorhabditis elegans* dystrophin-like gene *dys-1* lead to hyperactivity [64] and suggest a link with cholinergic nerve transmission [65]. Notably, we discovered three non-synonymous variants (SNP ChrZ: g.50362302 T > C causes p. 1142 Y > C; SNPs ChrZ: g. 50,363,889 A > C and ChrZ: g. 50,363,890 T > C cause p. 10,112 H > R; ChrZ: g. 50,399,919 G > A causes p. 74 H > Y) that showed extremely high degrees of genetic differentiation (F_ST_ values ranging from 0.90567 to 1) between the TW and C-L populations (Fig. 3D and E; Additional file 2: Table S7). *DYS* is reported to be closely related to the human disease Duchenne muscular dystrophy (DMD); muscle cells of DMD patients are abnormally fragile because of the lack of dystrophin [66]. The homologous gene in *S. mansoni* (*Smp_305490*) is specifically expressed in the muscles and nerves according to a previous study [67]. It has been reported that the *S. mansoni* dystrophin proteins bear multiple large insertions amounting to 100% of their expected size, especially in the C-termini, compared with those of other species [68]. We aligned the *S. japonicum* dystrophin (*Sj3555-RA*) protein sequence with the partial dystrophin protein sequences of other schistosomes (accession nos. DQ431250, EF120476, and EF12047) as well as two *C. elegans* dystrophin protein sequences (accession nos. F15D3.1 and NP_001293242.1) [68] and discovered that the *S. japonicum* dystrophin protein also had large insertions resembling those in *S. mansoni* (Fig. 3F, Additional file 1: Fig. S4). In addition, we observed two non-synonymous mutations (p. 1012 H > R; p.1142 Y > C) located near the C-terminus, and the mutations seemed to occur at conserved positions, especially p.1142 Y > C (Additional file 1: Fig. S4). Interestingly, these two missense variants exhibited different genotypes in TW from other populations and had the highest F_ST_ values (both F_ST_ values = 1) in the *DYS* genomic region (Fig. 3C and D). Previous research mentioned above provided evidence for the potential role of *DYS* in maintaining muscle or motor system homeostasis [66, 67]. In addition, dystrophin is required for organizing large acetylcholine receptor aggregates during muscle regeneration and is involved in cholinergic signaling [69]. The *DYS* gene in the TW population had strong positive selection signals, and specific non-synonymous mutations are likely to contribute to the biological functions associated with the slower development and milder pathological features of TW strains compared with other strains [16].

**Fig. 4:**
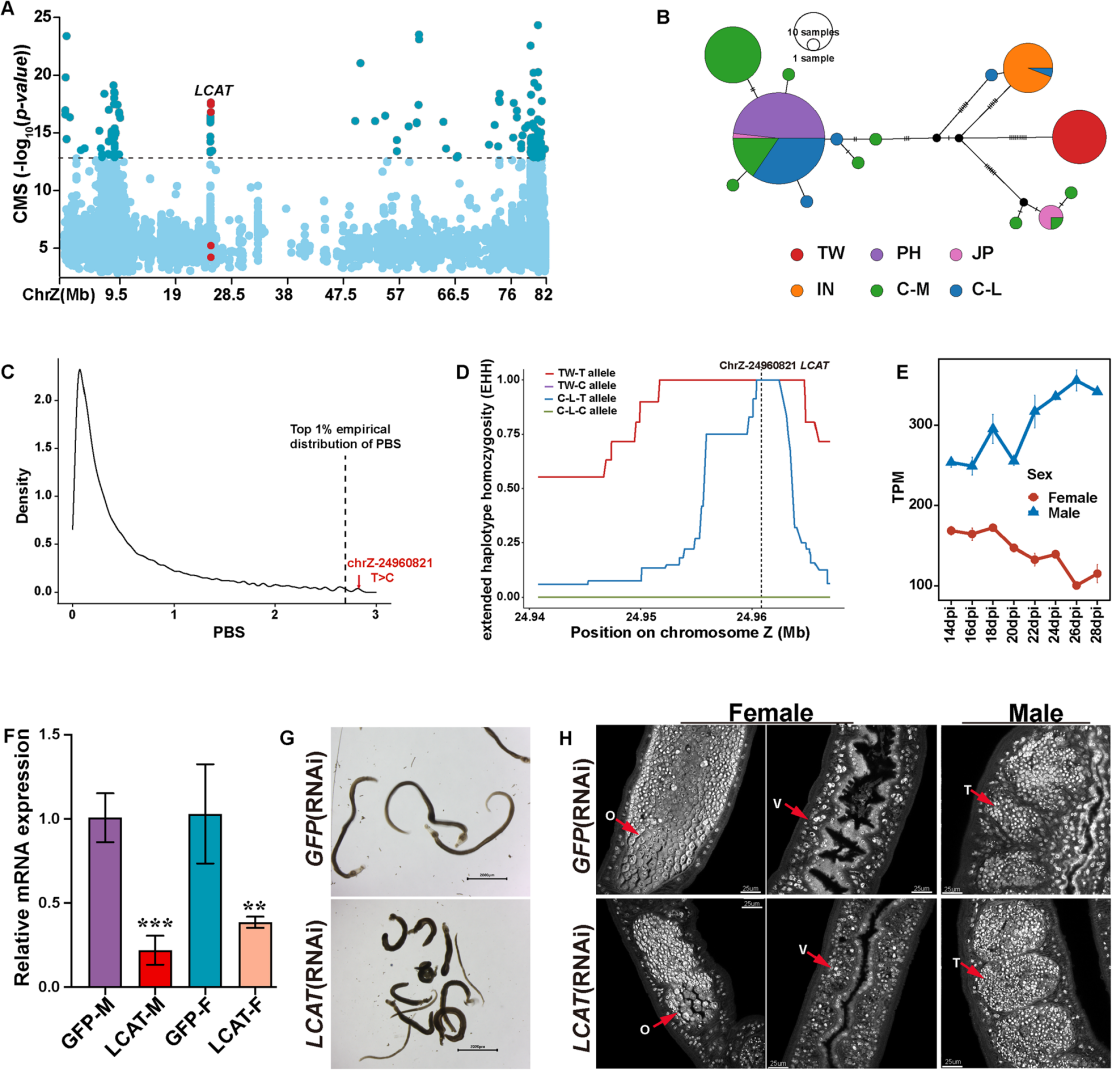
Candidate gene *LCAT* may be related to the slower development of *Schistosoma japonicum* in Taiwan compared with other regions. **A** Positively selected gene *LCAT* (*Sj2846*) identified by CMS analysis. The dashed lines represent the empirical thresholds for the selected region (top 1% empirical distribution of CMS score = 12.831). The genome region harboring the gene *LCAT* (lecithin-cholesterol acyltransferase) is marked in this figure. **B** Haplotype network based on the SNPs in the *LCAT* region (ChrZ:24,935,941–24,982,741). Each circle represents a haplotype, and its size suggests the number of individuals harboring the haplotype. **C** Kernel density distribution of the PBS statistic (top 1% value = 2.756928) for the entire Z chromosome in the TW sub-population. The dotted line marks an SNP site in *LCAT* (ChrZ-24960821). **D** Extended haplotype decay around the *LCAT-ChrZ-24,960,821* allele in the TW and C-L populations. **E** Relative mRNA expression levels of *LCAT* in females and males in developmental stages after infection of the definitive host. **F** Relative mRNA expression levels of *LCAT* in RNAi-treated parasites were analyzed by qPCR (mean ± standard error). *GFP* was used as the control group. Three biological replicates were performed. *P < 0.05, **P < 0.01, ***P < 0.001. **G** RNAi of *LCAT* causes parasite hypercontraction, Scale bar, 2000 μm. **H** Reproductive organs from *LCAT* and *GFP* RNAi parasites under confocal laser scanning microscopy. O, ovary; V, vitelline gland; T, testis. Three biological replicates were performed

Specifically, we found that the *LCAT* gene encoding lecithin-cholesterol acyltransferase exhibited significant signals of natural selection in the TW population (Fig. 4A). The region ChrZ: 24,960,000–24,975,000 harboring *LCAT* (*Sj2846*) showed the fourth-highest window score (see Methods) in the CMS (Additional file 2: Table S5). The corresponding maximum |iHS| and CMS scores were 4.981 and 17.631, respectively. The haplotype network analyses based on all identified SNPs in the *LCAT* gene region showed that TW individuals were clustered on an independent branch, suggesting a possible positive selection (Fig. 1B). Notably, we found one nonsynonymous variant (ChrZ: g. 24,960,821 T > C) exhibited a high PBS value (PBS value = 2.833934) in the *LCAT* gene region (Additional file 1: Fig. S5A), significantly higher than that of the entire Z chromosome (top 1% PBS value across the whole Z chromosome = 2.694496) (Fig. 4C). The extended haplotype homozygosity (EHH) surrounding this SNP revealed a pronounced signature of natural selection in the TW population (Fig. 4D). According to the longest transcript of the modified gene, this mutation site was located in exon 8 close to the 3ʹ end of *LCAT* and resulted in the substitution of isoleucine by threonine at the 447th amino acid position (Additional file 1: Fig. S5B), pointing to a potential functional role.

The gene *LCAT* encodes lecithin-cholesterol acyltransferase, which can convert free cholesterol into cholesteryl ester. The inability of schistosomes to synthesize fatty acids and sterols de novo makes it especially important to obtain lipid molecules in the establishment of schistosomes within their host [70, 71]. Moreover, previous research [72, 74] has indicated that schistosome egg embryonations acquire cholesterol esters from the host's high-density lipoproteins (HDL) in the bloodstream. Here, we demonstrate the expression profile of the *LCAT* gene during the development stages in mammalian hosts. Consistent with the findings of previous research, the male worm synthesized more lecithin-cholesterol acyltransferase (*LCAT*) before mating with females (Fig. 4E) [72, 73]. Interestingly, female worm maturation occurs between 22 and 28 days post-infection, during which the expression level of *LCAT* continues to increase in female worms, while it decreases in male worms [52]. Combined with previous studies, we suggest that *LCAT* may be involved in the process of female sexual maturity and egg embryonation and that higher concentrations of *LCAT* enzymes in males may contribute to enhancing female sexual maturation after mating.

To further investigate the function of *LCAT*, we performed RNA interference (RNAi) to perturb its expression in adult *S. japonicum*. After double-stranded RNA (dsRNA) treatment in vitro for 14 days, the relative mRNA expression of *LCAT* was knocked down by 94% in males and 80% in females compared with the *GFP*-treated (control) group (Fig. 4F). The knockdown of *LCAT* dramatically reduced parasite adherence to the substrate in vitro, and some coupled males and females were disengaged. Continuous interference treatment for 21 days resulted in the death of some worms (Fig. 4F). The same phenotype was also observed in *S. mansoni* in an earlier work when the *LCAT* homolog (*Smp_166500*) was knocked down in vitro [75]. In addition, dysplasia within the reproductive system was observed in *LCAT* RNAi female parasites. Compared with the control group, the diameters of ovaries were significantly reduced in the *LCAT* RNAi-treated female parasites, while the vitelline lobes were barely affected (Fig. 4H). No significant changes were observed in the testes of the *LCAT* RNAi-treated males (Fig. 4H). Therefore, we speculate that *LCAT* is involved in the process of female sexual maturity and that higher concentrations of *LCAT* enzymes in males may contribute to enhancing female sexual maturation after mating. A significant reduction in levels of serum lipids including cholesterol was observed in definite hosts infected with *S. mansoni* [76–78], possibly caused by changes in *LCAT* activity, synthesis, or secretion produced by the livers of infected animals [76]. Considering the zoophilic biological characteristics of the Taiwan population, our findings suggest that the *LCAT* gene in *S. japonicum* may play a crucial role in maintaining the maturation of the female reproductive organs in the definitive host, and the genomic mutations in *LCAT* may have contributed to the host-parasite co-evolution of the TW population.

Furthermore, we conducted a literature survey on the functions of the remaining 32 genes. GO enrichment results showed that gene karyopherin subunit alpha 4 (*KPNA4*, *Sj4228*) was involved in modulation by the virus of the host cellular process (GO:0019054, p = 3 × 10^–2^). The gene *Hmcn1* (*Sj4284*) encodes HEMICENTIN 1, which plays a crucial role in gonad development and gamete production in various model animals such as *C. elegans* and mice [75, 79–81]. In addition, we found that *Hmcn1* was highly expressed in sporocysts, a key stage for *S. japonicum* development in intermediate hosts (Additional file 1: Fig. S6A). During the portion of the life cycle spent in the definitive hosts, the expression level in male worms is at a relatively high level, while the expression level in female worms continues to decline (Additional file 1: Fig. S6B). Therefore, we hypothesized that the *Hmcn1* mutant in the TW population may lead to gonadal abnormalities or an increased probability of gamete error during meiosis or mating. These effects may be related to the weaker pathogenicity and longer incubation period of the TW strain [16].

### Genomic signatures of the intermediate host adaptation in the Z chromosomes of the mountain (C-M) population

The life cycle of *S. japonicum* depends critically on the parasite’s adaption to the local intermediate host *O. hupensis*. Differential compatibility has been reported between the snails and larvae from the Chinese mountain and lake populations [13]. We employed F_ST_, XP-EHH, iHS, and Tajima’s* D* to perform selective sweep scans to examine the potential genetic factors that may underlie the different compatibilities to intermediate hosts between mountainous (C-M) and lake (C-L) regions. A total of 342 candidate regions comprising 266 genes were identified (Additional file 2: Table S8). Gene ontology enrichment analysis revealed that the identified candidate genes were predominantly associated with protein serine/threonine kinase activity (GO:000467; *P* = 8.91 × 10^–3^), metallo-endopeptidase activity (GO:0004222; *P* = 2.24 × 10^–3^), and dynein light chain binding (GO:0045503; *P* = 1.39 × 10^–3^) (Additional file 1: Fig. S7; Additional file 2: Table S9).

**Fig. 5:**
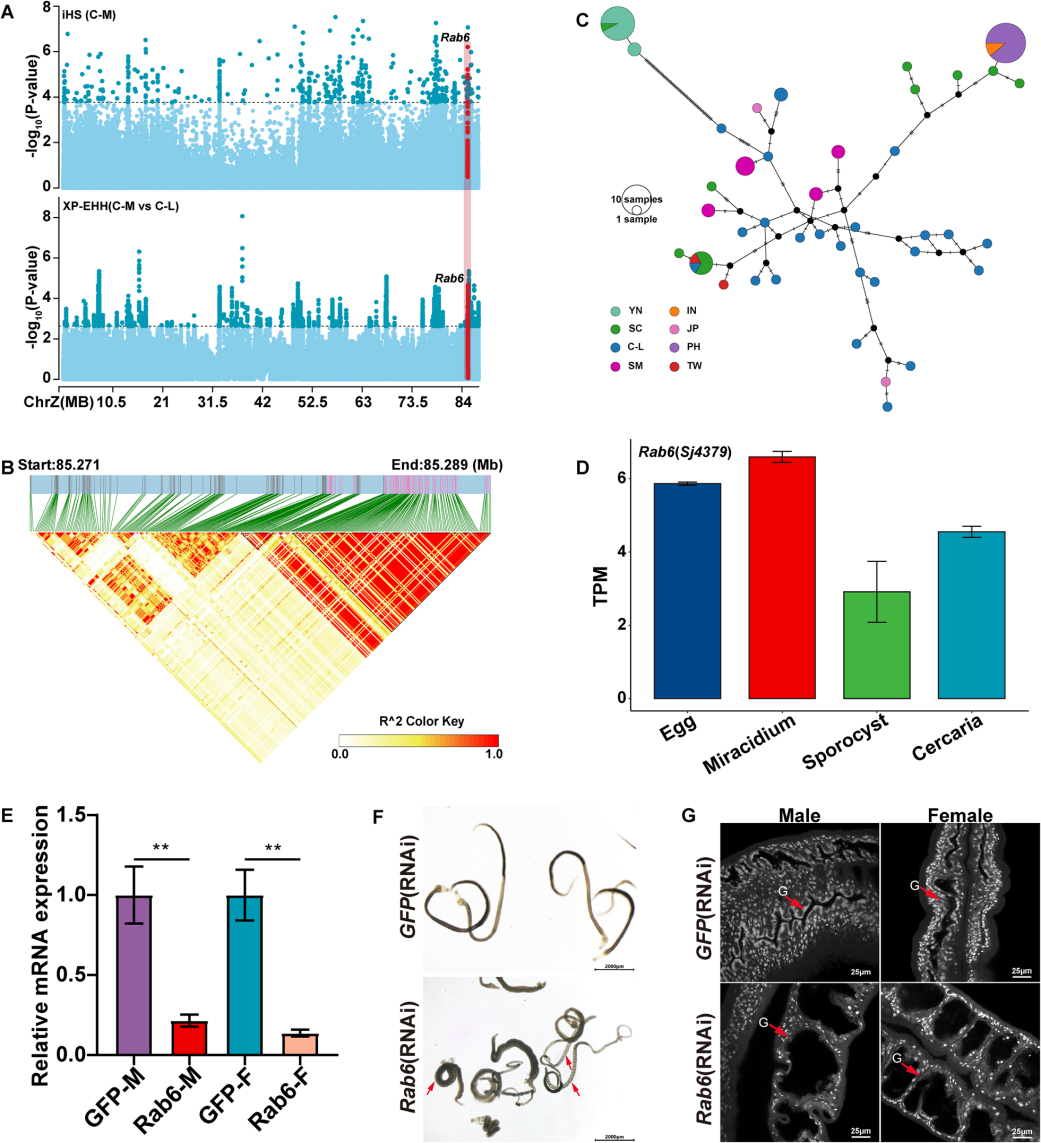
The candidate gene *Rab6* may be related to the compatibility of intermediate hosts of *Schistosoma japonicum* in the C-M strain. There are 13 samples from the C-M populations and 12 samples from the C-L populations. **A** Positively selected signatures identified by XP-EHH (between C-M and C-L populations; top 0.5% value = 2.64) and iHS (within the C-M population; top 0.1% *P* = 3.76). The dashed lines represent the empirical thresholds for the selected region. The candidate window harboring gene *Rab6* is highlighted in red. There are 13 samples from the C-M populations and 12 samples from the C-L populations. **B** Zoomed view of gene *Rab6* and a heatmap of linkage disequilibrium (LD) measured by the squared Pearson’s correlation coefficient (*r*^*2*^) for SNP variants. **C** Haplotype network based on 97 SNPs in the *Rab6* region. Each circle represents a haplotype, and its size suggests the number of individuals harboring the haplotype. **D** The relative mRNA expression levels of *Rab6* in the four larval stages, including egg, miracidium, sporocyst, and cercaria. **E** The relative mRNA expression levels of *Rab6* in RNAi-treated parasites were analyzed by qPCR (mean ± standard error). *GFP* was used as the control. Three biological replicates were performed. **F** RNAi of *RAb6* causes parasite hypercontraction, with the GFP-treated group as a control. Scale bar, 2000 μm. **G** The morphology of the control group and experimental group intestines under confocal laser scanning microscopy. Scale bar, 25 μm. The intestinal region has been highlighted with red arrows; G, gut. Three biological replicates were performed

Twenty overlapped candidate genes were screened by both XP-EHH and iHS (Additional file 1: Fig. S8A). We noted that the gene *Rab6* (*Sj4379*) was likely related to the incompatibility of the parasites with different intermediate hosts from the C-M and C-L regions (Additional file 2: Table S8; Additional file 2: Table S9). The *Rab6* gene encodes a Ras-related protein that is an evolutionarily conserved small GTPase [82]. It had strong selection signals in both iHS (maximum |iHS|= 4.9839196; ChrZ: g. 85,271,100) and XP-EHH (maximum XP-EHH = 4.246923; ChrZ: g. 85,273,170) (Fig. 5A). In addition, we checked the genomic characteristics of SNPs (XP-EHH > 2 and |iHS|> 2) in the *Rab6* gene region; most candidate SNPs were found in the non-coding region, with four SNPs discovered in the *Rab6* exon region. We observed an approximately 4-kb LD block (ChrZ:85,285,140–85,289,129, mean pairwise *r*^*2*^ = 0.88) near the 3ʹ end of the *Rab6* that contained 97 SNPs (Fig. 5B). The median-joining network analysis revealed that samples from the C-M population were classified into different branches, with two haplotypes (Hap 1 and 2) that were enriched in samples from Yunnan, while Sichuan-specific haplotypes were scattered into other branches (Fig. 5C). Among these 97 SNPs, we found that the derived alleles of 52 selected SNPs (|iHS|> 2 or XP-EHH > 2) were nearly fixed in the C-M population, particularly in the YN population, suggesting a significant selection event on the genomic region of *Rab6* (Additional file 2: Table S10). We observed that the *Rab6* gene in *S. japonicum* was more highly expressed in the miracidia, a key life stage in the snail intermediate hosts (Fig. 5D). *Rab6* is generally known to regulate retrograde transport in the Golgi body [83, 84] and is involved in a variety of other important cellular functions such as cell mitosis [85, 86] and innate immunity [82, 87, 88]. In the life cycle of *S. japonicum*, miracidia are hatched from the eggs in water and represent a vulnerable stage, as they are fully exposed to the external milieu and are required to counteract various environmental stresses, including pathogen infections [89]. Interestingly, *Rab6* shared 69.7% amino acid identity with *Rab-6.2* (the *Rab6* homolog in *C. elegans*, located on the X chromosome), a protein that is required for epidermal integrity and impermeability in *C. elegans*; *Rab-6.2* null mutants are more prone to rupture because of fragile cuticles [86]. In schistosomes, the epidermis is known as the tegument, and it is vital for food absorption and immunological evasion [90, 91]. We treated paired parasites for 30 days in vitro with *dsRNA* derived from *Rab6*; compared with the *dsGFP*-treated group, the *dsRab6*-treated group showed clear signs of abnormality, including curled bodies, a spongy appearance in parts of the parasite’s body, and visible swelling (Fig. 5D and E). Confocal laser scanning microscopy revealed that parasites in the experimental group had significantly enlarged intestines compared with those in the control group (Fig. 5F). Our findings showed that genetic mutations of *Rab6* may have had a key functional role in the adaptation of the mountain population, especially in Yunnan, to external conditions including intermediate host snails and pathogen stressors.

**Fig. 6:**
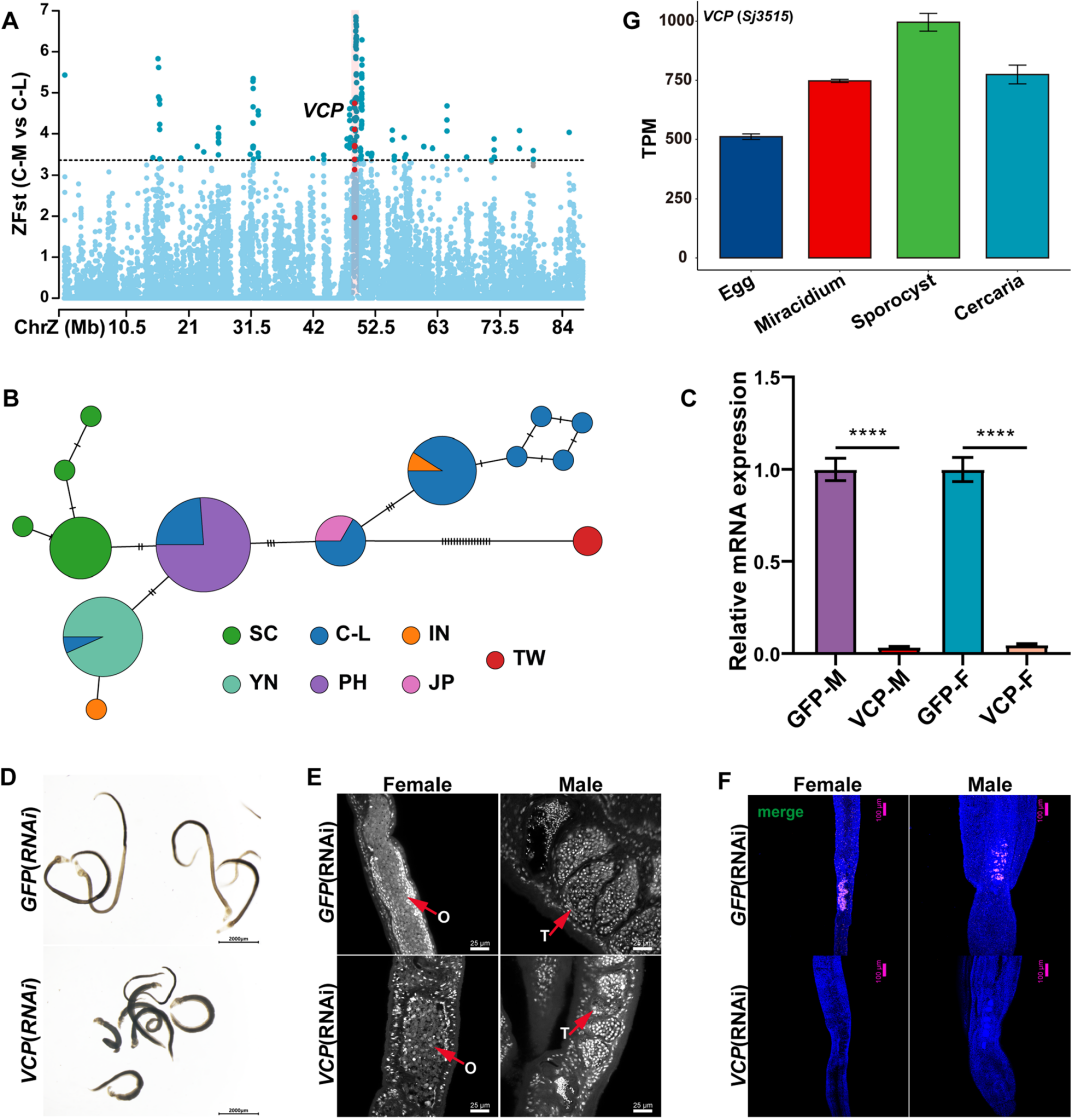
The candidate gene *VCP* identified by F_ST_ analysis may be related to the host immune evasion of *Schistosoma japonicum*. There are 13 samples from the C-M populations and 12 samples from the C-L populations. **A** Positively selected signatures identified by F_ST_ between the C-M and C-L populations. The dashed lines represent the empirical thresholds for the selected region. The candidate window harboring gene *VCP* is highlighted in red. **B** Haplotype network based on 27 SNPs in the *VCP* gene region (there are 27 SNPs in the whole VCP gene region of six populations). Each circle represents a haplotype, and its size suggests the number of individuals harboring the haplotype. **C** The relative mRNA expression levels of *VCP* in RNAi-treated parasites, with *GFP* as the control group, were analyzed by qPCR (mean ± standard error). Three biological replicates were performed. **D** RNAi of *VCP* causes parasite hypercontraction. The *GFP*-treated group was used as a control. Scale bar, 2000 μm. **E** Reproductive organs from *VCP* and *GFP* RNAi parasites under confocal laser scanning microscopy. O, ovary; T, testis. Three biological replicates were performed. **F** Edu labeling showing the expression of EdU^+^ proliferative cells (pink) in *GFP* (RNAi) and *VCP* (RNAi) parasites. Three biological replicates. **G** The relative mRNA expression levels of *VCP* in the four larval stages, including egg, miracidium, sporocyst, and cercaria

Among other selection signals, we identified the *VCP* (*Sj3515*), which may be involved in host selection and immune evasion. The *VCP* gene, also known as *P97*, which encodes Valosin-containing protein (*VCP*), stood out in the F_ST_ analysis (Fig. 6A). The protein is an AAA + ATPase that is both conserved and abundant, and it has been regarded as a host factor for a variety of pathogens, including parasites [92, 93]. The *VCP* gene exhibited different haplotypes in C-M and other populations (PH, C-L), and the haplotypes in the SC and YN were slightly different (Fig. 6B). Our interest in this gene stems from recent work showing that knocking down *SmP97* (*Smp_018240*) (the *S. mansoni* homolog of *VCP*) using RNA interference (RNAi) kills adult parasites in vitro and that *SmP97* RNAi-treated parasites in mice are quickly eliminated by the host immune system [75]. The amino acid sequence of *VCP* is 92.4% identical to *SmP97* (*Smp_018240*). Similar phenotypes were seen when RNAi treatment of *VCP* was applied to *S. japonicum* adults in vitro (Fig. 6C and D), suggesting that *VCP* may be conserved in schistosomes and is essential for the survival of *S. japonicum*. Furthermore, we found that parasites knocked down for *P97*/*VCP* using 5-ethynyl-2-deoxyuridine (EdU) labeling showed decreases in germ cell development, particularly in spermatocytes and oocytes (Fig. 6E and F). Our transcriptomic analysis showed that the sporozoites had much higher *VCP* gene expression levels than other larval stages (Fig. 6G). This suggests that *VCP* not only functions in host immune evasion in the final host mammal but also plays a key role in the establishment of schistosomiasis in the intermediate host. However, the mechanism of *VCP*/*P97* in schistosomiasis and intermediate host snails is not yet fully understood, although we speculate that it may be related to host immune evasion or adaptation. Further research is needed to elucidate the precise role of *VCP*/*P97* in the pathogenesis of schistosomiasis.

Another gene, *Nep4* (*Sj4380*), was selected by both XP-EHH and iHS (Additional file 1: Fig. S8C), and it encodes a metallopeptidase named Neprilysin4 (Additional file 2: Table S9; Additional file 1: Fig. S7) that is thought to aid the parasite in escaping the immune attack of *O. hupensis* after invasion by miracidia [53]. *Nep4* was highly expressed in the miracidium, a critical offstage in the invasion of intermediate hosts (Additional file 1: Fig. S8B). We detected an approximately 900-bp genomic segment with strong positive selection signals located at 600 bp upstream of the 5’ end of gene *Nep4* (85,313,239 bp) (Additional file 1: Fig. S8D and E). This segment was detected by both XP-EHH and iHS, indicating its potential involvement in positive selection.

## Discussion

Due to greater knowledge about the parasite, heightened awareness, and a strong focus on schistosomiasis eradication efforts in many countries, *S. japonicum* has almost been eliminated in Japan and China, with just a few isolated instances being documented. The DNA samples collected by Luo et al. from wild strains in mainland China, Taiwan, the Philippines, and Indonesia represent the most comprehensive genomic data of *S. japonicum* to date [16]. Using SNP data on autosomes, researchers investigated the genetic diversity and population structure of *S. japonicum* and identified the genomic basis of host-switching. However, the Z chromosome of *S. japonicum* contains genes that play crucial roles in parasite development, reproduction, and sex determination [94]. Owing to the smaller effective population size (*Ne*) [95], sex chromosomes (X/Z chromosomes) are thought to be more sensitive to genetic drift and experience stronger selection pressure than autosomes [96]. In this study, we considered the Z chromosome and analyzed the SNP data to better understand the population structure and local adaptations of *S. japonicum*. We found a faster evolution rate on the Z chromosome of *S. japonicum* than on the autosomes, potentially attributed to stronger selection pressures on the Z chromosome. However, although the effect of adaptive evolution was statistically significant, it was insubstantial in magnitude compared to the variation among genes. Moreover, because our study was focused on adaptive evolution, we did not completely rule out the effects of genetic drift. In addition, we employed a battery of complementary statistical approaches to detect positive selection, including F_ST_, Tajima’s *D*, XP-EHH, iHS, and CMS to gain a deeper understanding of genomic signatures of the Z chromosome that may be associated with different phenotypic traits. Such knowledge will provide a more comprehensive insight into the genomic mechanisms underlying the genetic factors involved in host-parasite adaptation.

Compared with previous studies based on mitochondrial and autosome DNA data [9, 10], the population structure revealed by the SNPs of the Z chromosome divided 72 *S. japonicum* samples into six subgroups, and the largest difference was the substructure in the mountain population being more directly distinguished in both PCA and admixture analysis based on Z chromosome data. The substructure of mountain populations was revealed at the sex chromosome level. In the C-M population, we detected several genes that may be related to host immune responses, including *Rab6*, *Nep4*, and *VCP*. These genes are highly expressed in the larval stages and are likely to be associated with the invasion of the intermediate hosts. For instance, *Rab6* showed a strong selection signal in the C-M population. In vitro* Rab6* treatment with dsRNA caused aberrant phenotypes in the parasite, including a curled body and a spongy appearance. Interestingly, snails from the Yunnan and Sichuan provinces grouped in separate clades on the phylogenetic tree [97], a result that may be related to the difference in *Rab6* haplotypes between the two populations. Besides, *Nep4* encodes Neprilysin4, a protein that may be involved in the inactivation of the snail host’s immunocytes [53]. RNA-seq data showed that *Nep4* was highly expressed at the miracidia stage. Another gene, *VCP*, has been reported to assist *S. mansoni* in evading the immune attack of the definitive host [75]. RNAi treatment of *VCP* on paired adult *S. japonicum *in vitro resulted in phenotypes similar to *S. mansoni*. *Nep4* and *VCP* are crucial for the establishment of the parasite in *O. hupensis*, whereas *Rab6* is required for egg hatching and miracidia resistance to the environmental immunological response. Differential compatibility has been reported between the snails and larvae from the Chinese mountain and lake populations [13]. The genetic variations of these genes may have contributed to the adaptive evolution of *S. japonicum* in response to mountain environments and intermediate hosts in the C-M population. Moreover, these gene differentiations and sub-structures observed between the YN and SC sub-populations suggest a more complicated co-evolutionary history of *S. japonicum* in these mountain areas.

The Taiwan population cannot parasitically reproduce in humans; it exhibits zoophilia and has weaker pathogenicity [5]. When comparing autosomal and sex (Z) chromosomal genetic diversity, we observed the largest Dxy, and the lowest diversity in the Z chromosome occurred in the TW population. This suggested potential local adaptation. We identified 34 selected candidate genes in the Taiwan population associated with cellular processes such as DNA replication, lipoprotein metabolism, and modulation by symbionts of the host. Among these, the gene *DYS* (*Sj3555*) associated with cholesterol transport and *LCAT* (*Sj2846*) relevant to acetylcholine transmission ranked first and fourth, respectively, in the CMS window scores in the TW population. RNAi experiments suggested the potential function of *LCAT* in the reproduction, development, and survival of *S. japonicum* in the definitive host. *DYS* is closely related to muscle and nerve development, and *DYS* mutants have been reported to be associated with increased levels of acetylcholine [64]. Schistosomes are entirely reliant on their definitive host blood for essential nutrients, including cholesterol and glucose. *LCAT* and *DYS* may play important roles in the transport of cholesterol and glucose from host blood into schistosomes [98, 99]. Regarding the zoophilia and low pathogenicity to mammalian hosts of the TW population, our findings suggest that the positive selections on the Z chromosome may have contributed to the host-parasite co-evolution.

## Conclusions

In conclusion, we detected potential selected regions on the Z chromosome of *S. japonicum* and further identified candidate genes associated with geographically specific phenotypes, host immune responses, and environmental conditions. Our study has contributed to the understanding of adaptive evolution in response to hosts and environmental factors in *S. japonicum* at the sex chromosome level. Overall, these findings broaden our understanding of the complex interplay between hosts and parasites and highlight the importance of considering both biological and ecological factors in the study of parasitic infections.

### Supplementary Information

**Additional file 1: **Fig. S1–S8

**Additional file 2: **Table S1 to S11.

## Data Availability

The whole genome re-sequencing data of 72 *Schistosoma japonicum* has been deposited in the NCBI (project ID: PRJNA789681). The chromosome-level reference genome of *S. japonicum* (SjV3) was download from Zenodo (https://zenodo.org/record/5795038). Four larval stage transcriptomes of *S. japonicum* were downloaded from NCBI (project ID: PRJNA719283; https://www.ncbi.nlm.nih.gov/bioproject/PRJNA719283). Developmental stages transcriptomes of adult *S. japonicum* were downloaded from (project ID: PRJNA343582; https://www.ncbi.nlm.nih.gov/bioproject/PRJNA343582). Five re-sequenced *Schistosoma mansoni* data were obtained from the European Nucleotide Archive (ENA) (SRA accession numbers: SRR13624153, SRR13624155, SRR13624156, SRR13624157, and SRR13624158). This paper does not report the original code.
