# Supplementary material for: Characterizing genetic variation on the Z chromosome in Schistosoma japonicum reveals host-parasite co-evolution
Source: Parasit Vectors. 2024 May 8;17:207. doi: 10.1186/s13071-024-06250-4 (PMC11080191; doi:10.1186/s13071-024-06250-4)
Supplement: Supplementary file 1 — Additional file 1: Fig. S1–S8 [file 13071_2024_6250_MOESM1_ESM.pdf]

Additional file 1

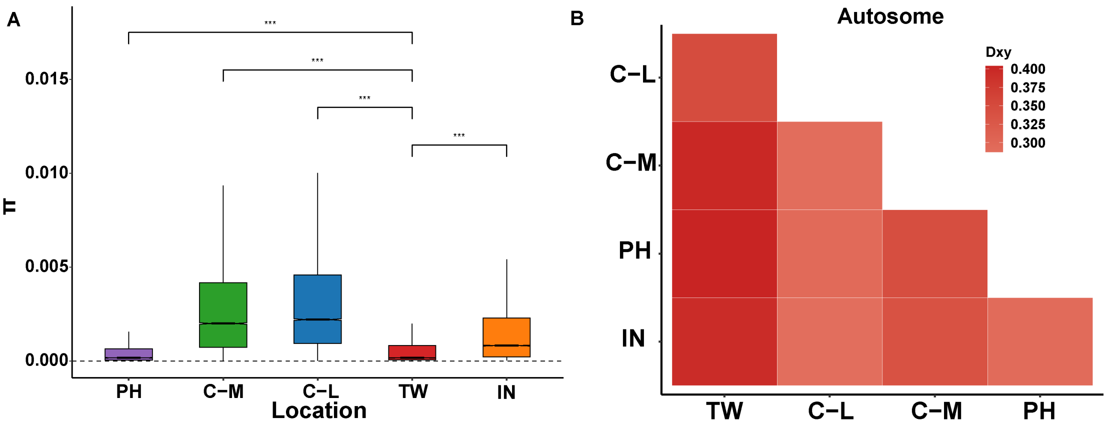

Fig S1. Genetic diversity in six *S. japonicum* sub-populations based on variants on the Z chromosome relative to Fig 1.

(A and B) Nucleotide diversity (π) and Tajima's D in five *S. japonicum* sub-populations based on variants of the Z chromosome. Student's t-test was used to compare the differences between populations. *, *P*<0.05, **, *P*<0.01, ***, *P*<0.001.

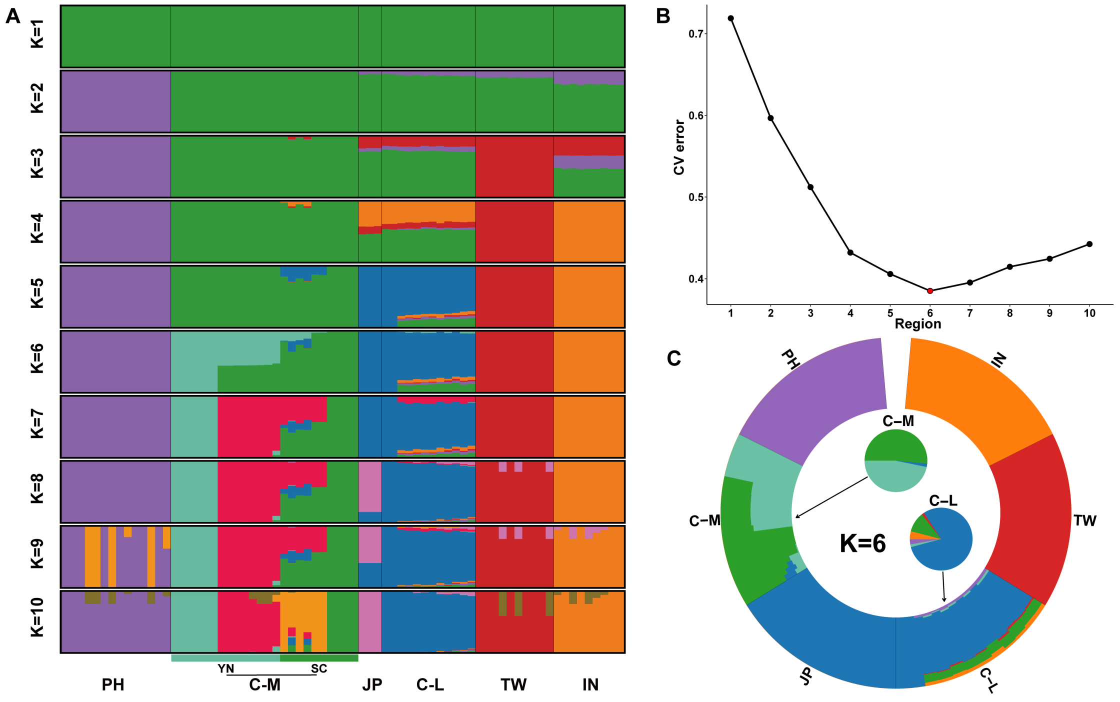

Fig S2. Population structure of *S. japonicum*. Related to Fig 2.

(A) Population structure revealed by admixture with K from 1 to 10.

(B) Cross-validation (CV) error plot for admixture analysis. We chose K=6 to analyze the SNP data, as this was the value that minimized the error.

(C) Donut pie chart of population genetic structure with the best K=6. We show pie charts for groups with complex ancestry components in the center of the circle chart.

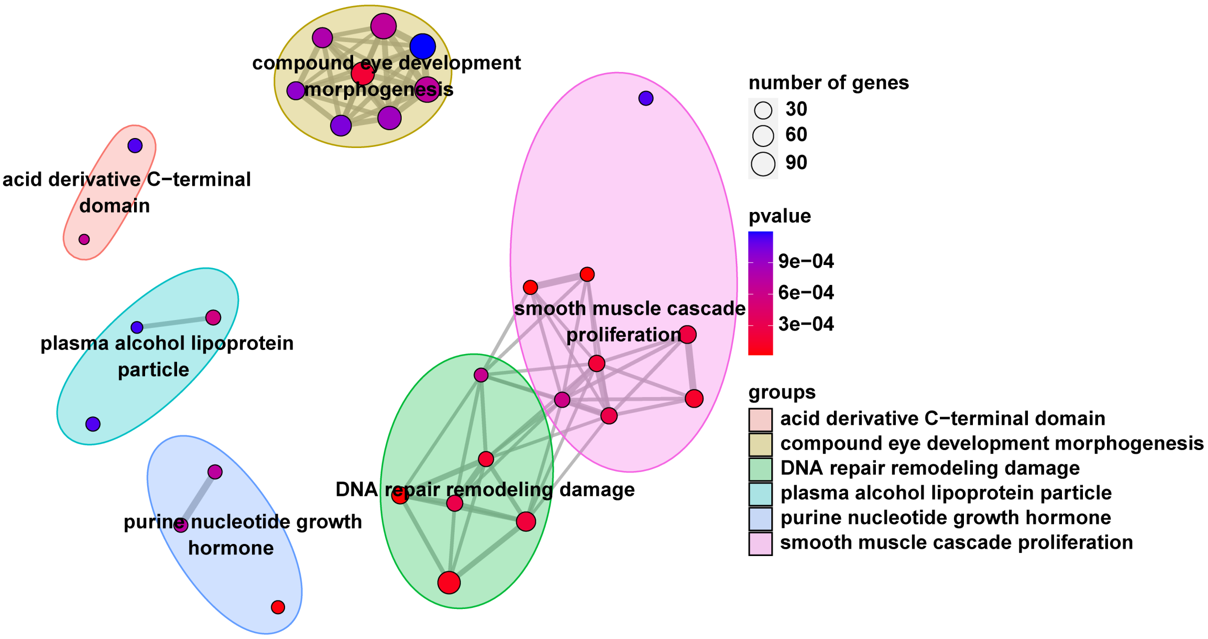

Fig S3. Gene ontology (GO) enrichment of 2,116 genes on the Z chromosome of *S. japonicum.*

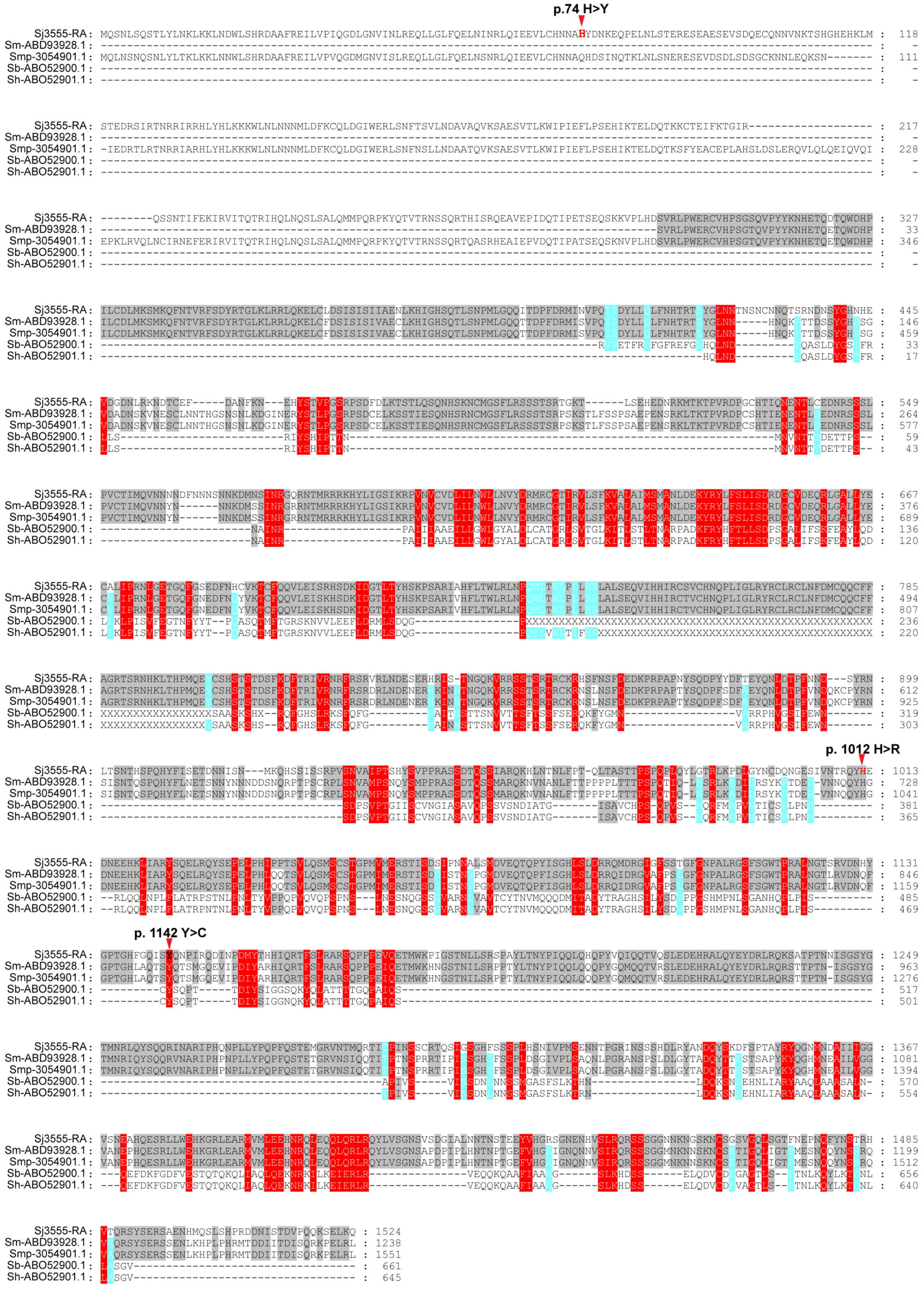

Fig S4. Alignment of full-length *S. japonicum* dystrophin (*Sj3555-RA*) with *S. mansoni* (*Sm*), *S. bovis* (*Sb*), and *S. haematobium* (*Sh*) dystrophin C-termini. Alignment was performed using CLUSTALW. Related to Fig 3.

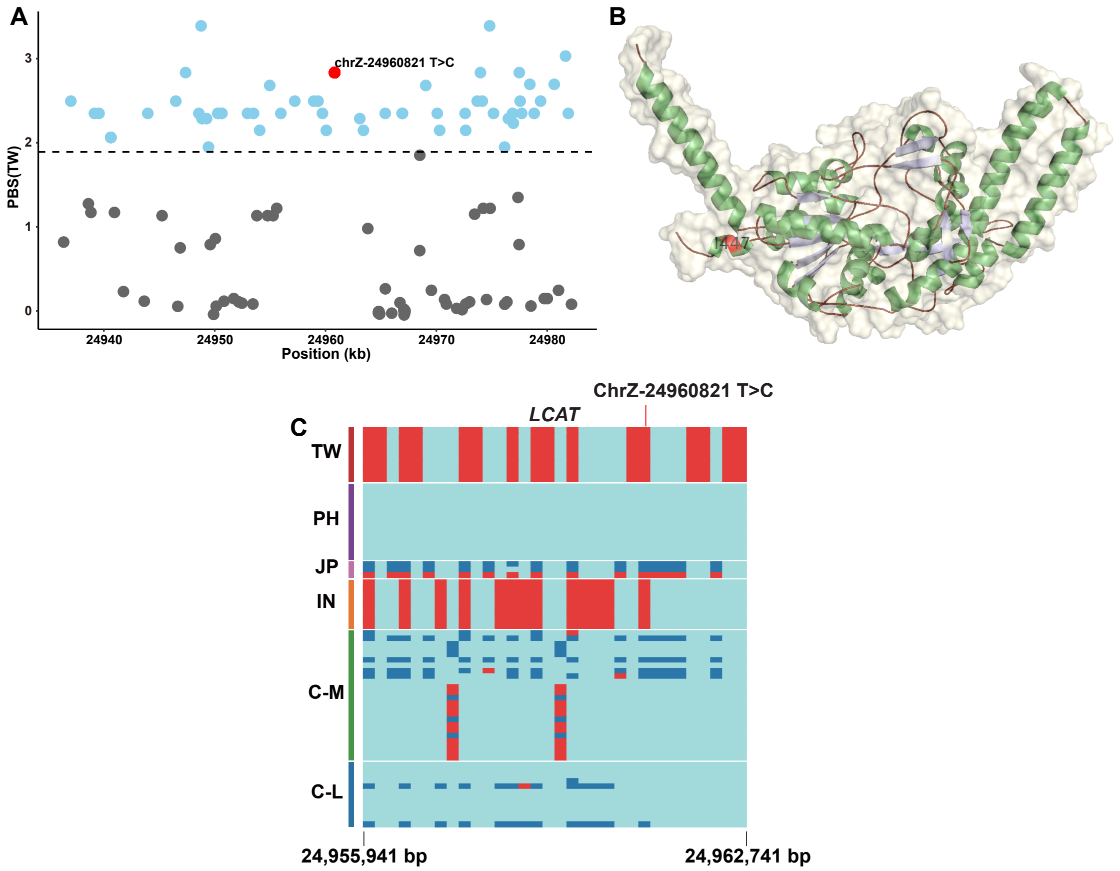

Fig S5. Positively selected signatures between TW and C-L populations identified by CMS. Related to Fig 4.

(A) PBS statistics over the *LCAT* region. The site of *LCAT*-ChrZ-24960821 is highlighted in red. The dotted line represents the top 10% threshold (top 1% PBS value = 2.469032)

(B) Protein structure modeling of *LCAT*. Non-synonymous mutations that are highly differentiated between the TW and C-L populations are highlighted in red.

(C) Haplotype heatmap of SNPs around the *LCAT* in six *S. japonicum* sub-populations. The site of *LCAT*-ChrZ-24960821 is marked with a label.

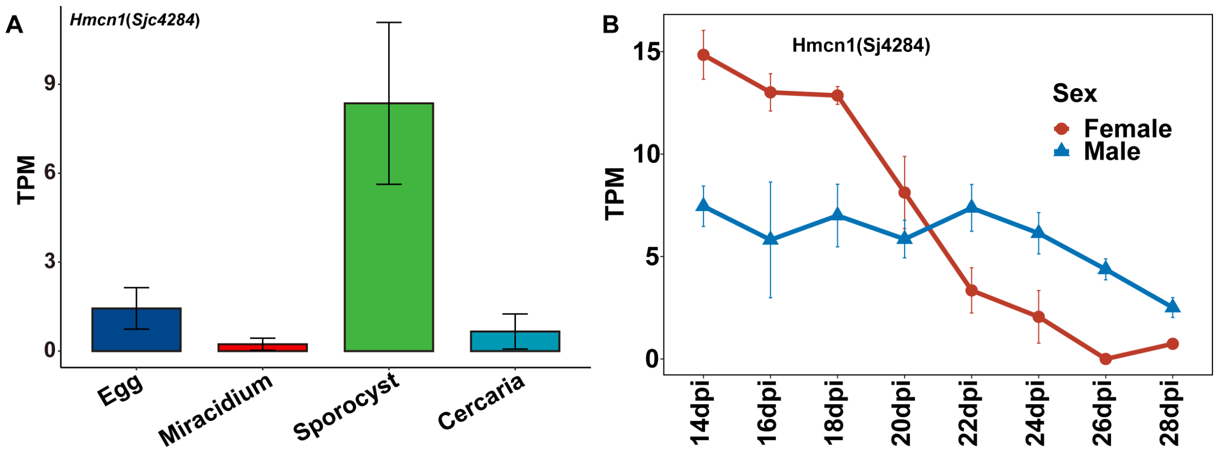

Fig S6. Relative mRNA expression levels of *Hmcn1* (Hemicentin 1; *Sj4284*) selected in TW populations

Relative mRNA expression levels of *Hmcn1* in the free life period (A) and of female and male in developmental stages after infection of the definitive host (B).

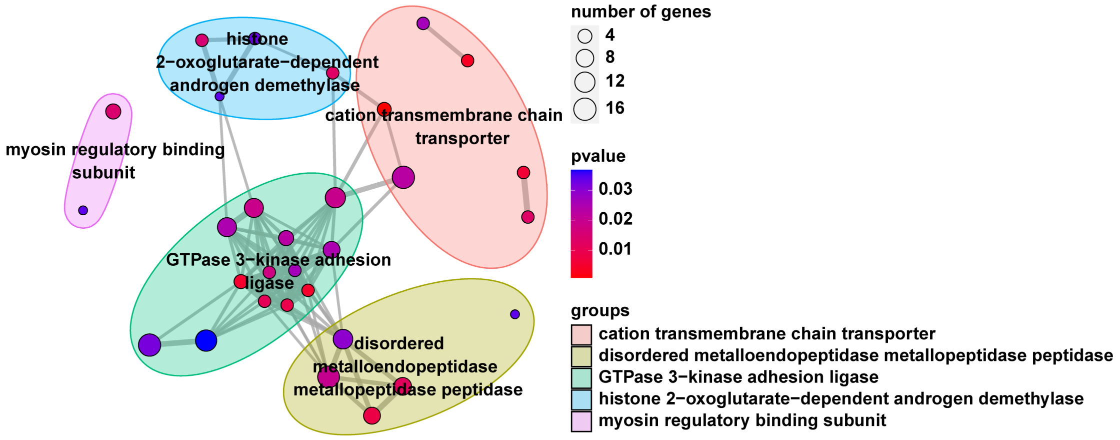

Fig S7. GO enrichment of the selected genes in the C-M population identified by four methods (FST, XP-EHH, Tajima's *D*, and iHS) (*P-value* < 0.01).

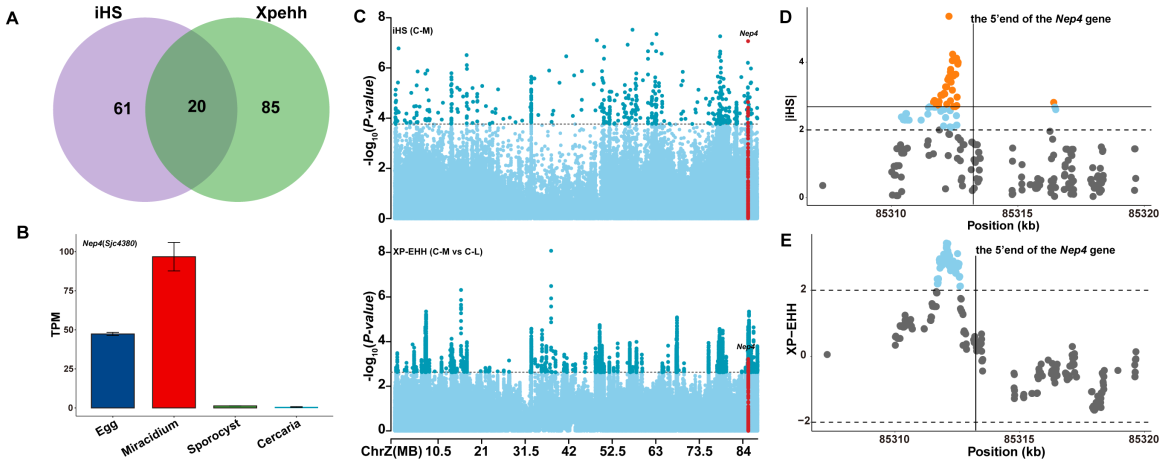

Fig S8. The candidate gene *Nep4* may be related to the compatibility of intermediate hosts of *S. japonicum* in the C-M strain.

(A) Venn diagram of candidate genes identified by XP-EHH (between C-M and C-L populations, top 0.5% value = 2.64) and iHS (within the C-M population, top 0.1% value = 3.76).

(B) The relative mRNA expression levels of *Nep4* in the four stages (egg, miracidium, sporocyst, and cercaria).

(C) Positively selected signatures for *Nep4* identified by XP-EHH and iHS. The dashed lines represent the empirical threshold for the selected region. The candidate genes are highlighted in red.

(D) The |iHS| values corresponding to selected regions containing the gene *Nep4* on the Z chromosome. The horizontal black dotted line represents |iHS| = 2, and the horizontal black solid line represents the top 1% |iHS| value.

(E) The XP-EHH values corresponding to selected regions containing the gene *Nep4* on the Z chromosome. The upper and lower horizontal black dotted lines represent 2 and —2, respectively.
